# Association between Cardiovascular Disease and Liver Disease, from a Clinically Pragmatic Perspective as a Cardiologist

**DOI:** 10.3390/nu15030748

**Published:** 2023-02-01

**Authors:** Mitsutaka Nakashima, Kazufumi Nakamura, Takahiro Nishihara, Keishi Ichikawa, Rie Nakayama, Yoichi Takaya, Norihisa Toh, Satoshi Akagi, Toru Miyoshi, Teiji Akagi, Hiroshi Ito

**Affiliations:** Department of Cardiovascular Medicine, Graduate School of Medicine, Dentistry and Pharmaceutical Sciences, Okayama University, Okayama 700-8558, Japan

**Keywords:** liver disease, heart failure, atherosclerotic cardiovascular disease, non-alcoholic fatty liver disease

## Abstract

Cardiovascular diseases and liver diseases are closely related. Non-alcoholic fatty liver disease has the same risk factors as those for atherosclerotic cardiovascular disease and may also be a risk factor for atherosclerotic cardiovascular disease on its own. Heart failure causes liver fibrosis, and liver fibrosis results in worsened cardiac preload and congestion. Although some previous reports regard the association between cardiovascular diseases and liver disease, the management strategy for liver disease in patients with cardiovascular diseases is not still established. This review summarized the association between cardiovascular diseases and liver disease. In patients with non-alcoholic fatty liver disease, the degree of liver fibrosis progresses with worsening cardiovascular prognosis. In patients with heart failure, liver fibrosis could be a prognostic marker. Liver stiffness assessed with shear wave elastography, the fibrosis-4 index, and non-alcoholic fatty liver disease fibrosis score is associated with both liver fibrosis in patients with liver diseases and worse prognosis in patients with heart failure. With the current population ageing, the importance of management for cardiovascular diseases and liver disease has been increasing. However, whether management and interventions for liver disease improve the prognosis of cardiovascular diseases has not been fully understood. Future investigations are needed.

## 1. Introduction

Recently, a strong association has been shown between cardiovascular disease (CVD) and liver disease. Not only do these diseases have several similar risk factors such as dyslipidemia, diabetes mellitus, and obesity, but also atherosclerotic cardiovascular disease (ASCVD) progresses in accordance with worsening metabolic conditions in patients with non-alcoholic fatty liver disease (NAFLD) [1,2,3,4,5,6]. Additionally, cirrhosis is reported to further impair natriuresis, which might cause increased cardiac preload and congestion in patients with heart failure (HF), cardiac dysfunction, and even pulmonary hypertension due to portal hypertension [7]. On the contrary, chronic HF may lead to liver damage owing to liver congestion, which eventually leads to liver fibrosis and cirrhosis. Furthermore, several drugs which are widely used for CVD have been also reported to possibly cause liver damage and steatosis [8,9,10].

Considering these interactions between CVD and liver disease, the evaluation of both diseases is important in the current ageing era. Although there are some previous reports that regard the association and interaction between CVD and liver disease, the evaluation and management strategy for liver conditions in patients with CVD are not still established. Thus, in this review, we summarized the current evidence supporting the association between CVD and liver disease, focusing on the relationship between ASCVD with NAFLD and the interaction between HF and liver dysfunction.

## 2. The Interlink Connections of ASCVD and NAFLD

In patients with NAFLD, CVD is the most important comorbidity as well as liver-associated complications. CVD is the most common cause of death in these patients [11,12,13]. ASCVD and NAFLD share the same risk factors, such as dyslipidemia, diabetes mellitus, and obesity; however, NAFLD itself is considered a promoting factor for ASCVD [1,2,3,4,5,6]. NAFLD is associated with dyslipidemia, oxidative stress, microbiome disturbances, chronic inflammation, insulin resistance, coagulation disorders, and endothelial dysfunction, and these factors might also be associated with CVD [14,15]. Owing to these factors, NAFLD has a strong association with prognosis in CVD.

**Figure 1:**
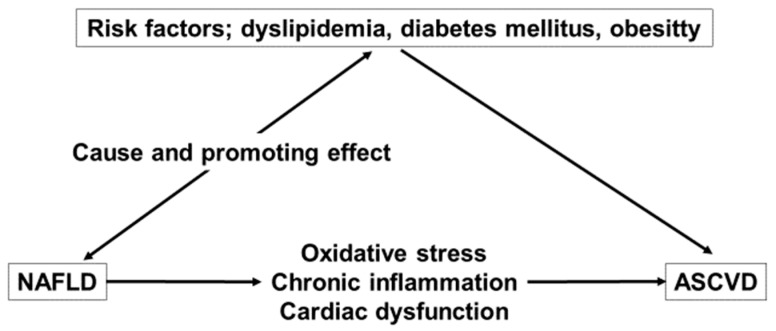
The relationship between NAFLD and ASCVD. NAFLD, non-alcoholic fatty liver disease; ASCVD, atherosclerotic cardiovascular disease.

Figure 1 shows a summary of the relationship between CVD and NAFLD.

### 2.1. Dyslipidemia

NAFLD is associated with abnormal lipid metabolism and altered glucose metabolism. Patients with NAFLD tend to have high triglyceride concentrations, high low-density lipoprotein concentrations, oxidated low-density lipoprotein and remnant cholesterol, and low high-density lipoprotein concentrations, which are established risk factors for ASCVD [6,16,17,18]. Dyslipidemia can become one of the causes of NAFLD and also be worsened by NAFLD. Elevated plasma insulin and glucose levels, which are caused by diabetes mellitus and insulin resistance, and excess plasma lipid lead to increased de novo lipogenesis and decreased intracellular triglyceride hydrolysis. This increases hepatic triglyceride content, which causes increased plasm triglyceride levels. In addition, increased intracellular cholesterol in the liver reduced membrane-bound low-density lipoprotein receptor and low-density lipoprotein uptake to the liver. These changes contribute to increased large very-low-density lipoprotein 1 and small dense low-density lipoprotein, which finally accelerate atherosclerosis [19]. Notably, the Mediterranean diet and antioxidant formulation efficiently improve anthropometric parameters, lipid profile, and insulin sensitivity and reduce hepatic fat accumulation and liver stiffness in patients with NAFLD as preventive management [20]. Because the therapeutic strategy for NAFLD has not been established yet, diet therapy may be one of the few efficient therapies for patients with NAFLD.

### 2.2. Oxidative Stress and Chronic Inflammation

NAFLD is associated with oxidative stress and chronic inflammation, which are strongly associated with CVD [14,15]. Insulin resistance is not only a major pathogenic factor associated with NAFLD but also causes impaired glucose transport into skeletal muscle and uninhibited lipolysis in adipose tissue [21,22]. This leads to hepatic-derived oxidative stress via reactive oxygen species, such as super oxides. Oxidative stress leads to proinflammatory cytokine expression [14,22,23,24]. Tumor necrosis factor-α mRNA is overexpressed in both the liver and adipose tissue and has a relationship with liver fibrosis stages, and toll-like receptor 2 activates the inflammasome in the development of NAFLD [23,24]. Additionally, obesity and metabolic dysregulation, which are also involved in the pathogenesis of NAFLD, are associated with alterations in the gut microbiome [25]. These alterations cause increased fatty acid absorption and endotoxin penetration, which further increase the inflammatory response. Because the progression of both ASCVD and HF have relationships with oxidative stress and inflammation, these oxidative stressors and the resulting inflammation can be promoting factors in the progression of CVD [26]. In addition, recent studies indicate that immune cells significantly affect the development of HF. Immune cells such as cluster of differentiation 4 T cells or cluster of differentiation 8 T cells have potential impacts on the development or regulation of HF and cardiac remodeling in heart ischemia [27,28,29,30,31].

### 2.3. Coagulation Disorders and Endothelial Dysfunction

NAFLD is also associated with coagulation disorders and endothelial dysfunction. The alternation of the synthesis of coagulation factors in the liver causes coagulation disorders in patients with chronic liver diseases [32]. Although both prolonged bleeding and hypercoagulation can appear according to the balance of procoagulant and anticoagulant factors, those changes tend to proceed simultaneously, and their balance is restored and rebalanced in most patients with cirrhosis [32,33]. However, in patients with NAFLD, the frequency of both arterial and venous thrombotic events is increased compared with the absence of NAFLD, owing to hypercoagulation [15,34]. Increased platelet activation and activity of procoagulant factors and hypo fibrinolysis are observed in patients with NAFLD. The changes in the plasma procoagulant profile, such as increased circulating procoagulant factors of VIII, IX, XI, von Willebrand factor, and fibrinogen occur in patients with NAFLD owing to obesity, insulin resistance, and chronic inflammation [35,36,37]. In addition, these coagulation disorders with hypercoagulation proceed as the severity of NAFLD increases [38,39,40]. The oxidative stress and inflammation, that are also associated with NAFLD as above mentioned, also cause endothelial vascular dysfunction [41,42], and these changes can also cause CVD in patients with NAFLD [43].

## 3. The Interactions between HF and Liver Dysfunction

Most patients with HF have non-cardiovascular comorbidities [44]. Because these comorbidities have a significant impact on the prognosis in patients with HF, the management of comorbidities in patients with HF is recommended in current guidelines for the management of HF. It is well-known that hemodynamic abnormalities in patients with both acute and chronic HF cause liver damage [45,46,47]. Hepatic congestion due to elevated central venous pressure leads to cardiogenic liver injury in chronic HF, and in acute HF, acute liver damage is led by impaired liver perfusion. This results in insufficient perfusion to meet the metabolic demands of hepatic cells, as well as liver congestion [47,48]. In liver congestion, pressure in the central veins of the hepatic lobules increases, and bile ducts are compressed by increased hydrostatic pressure, which causes the explosion of poorly oxygenated blood to extensive areas around the central veins extending to the adjoining central veins and the cholestasis, and these conditions lead to the elevation of aminotransferase levels, hepatocellular dysfunction, centrilobular necrosis, and finally, liver fibrosis and cirrhosis [45,47,49]. In animal studies, the response of natriuresis regulated by cyclic adenosine monophosphate is impaired in a rat model of cirrhosis with ascites because of their liver dysfunction [50]. Extracellular cyclic adenosine monophosphate is a second messenger that is produced in the liver and regulates sodium reabsorption in the proximal renal tubules [51]. These results may indicate that liver disorder links impaired natriuresis, which can increase cardiac preload and organic congestion. Furthermore, cirrhosis is reported to worsen ventricular hypertrophy, left ventricular diastolic dysfunction, and left ventricular systolic dysfunction [46]. The specific feature of hemodynamics in patients with cirrhosis is that cardiac output increases due to splanchnic arterial vasodilation and reduced systemic vascular resistance [7]. Although the reduced systemic vascular resistance tends to preserve high cardiac output, cardiac dysfunction may supply insufficient blood perfusion to meet systemic organ demands, which leads to hyperdynamic unloaded HF [46]. In addition, the electrocardiographic QT interval will be also prolonged in patients with cirrhosis [52]. These hemodynamic and electrocardiographic changes are caused by various mechanisms such as abnormality of sympathetic nervous activity, alteration at the β-receptor, altered collagen structure, and endotoxins or cytokines such as the tumor necrosis factor-α (TNF-α) [46]. These findings show that HF and liver dysfunction are associated and that these conditions interact and may create a vicious cycle.

Additionally, liver dysfunction is one of the most important complications in adults after the Fontan procedure, which is a specific operation for patients with a functional single ventricle [53]. In Fontan circulation, continuous central venous hypertension owing to the absence of the sub-pulmonary ventricle causes a specific congestive hepatopathy known as Fontan-associated liver disease as a long-term complication [53]. Fontan-associated liver disease often causes cirrhosis and hepatocellular carcinoma, which are associated with a worse clinical prognosis [54]. The terminal stage of Fontan-associated liver disease can even develop hepatic encephalopathy [55]. Thus, regular liver imaging and liver function tests are recommended after the Fontan procedure [56]. After the development of cirrhosis and portal hypertension due to Fontan-associated liver disease, intrinsic substances such as nitric oxide increase and lead to systemic vasodilation [57]. This abnormal systemic vasodilation cause inappropriately reduced systemic vascular resistance and systemic blood pressure and causes end-stage heart failure with inappropriately high cardiac output. This abnormal hemodynamic situation is known as a sign of an extremely worse prognosis. In the prevention of the progression of Fontan-associated liver disease, the maintenance of central vein pressure at a lower level is recommended [58].

As a clinically specific cause of heart failure caused by liver disease, porto-pulmonary hypertension is one of the important causes of right-sided heart failure. Approximately 1–2% of patients with portal hypertension due to liver diseases develop porto-pulmonary hypertension [9,10]. Patients who are candidates for trans jugular portosystemic shunting or liver transplantation have a higher risk of porto-pulmonary hypertension. Although the mechanism of development for porto-pulmonary hypertension has not been fully elucidated yet, the presence of portal hypertension rather than the severity of liver disease has been considered to play a central role in the development of porto-pulmonary hypertension [59,60,61]. Because the prognosis of porto-pulmonary hypertension is poor unless treated, patients with portal hypertension need to be screened for pulmonary artery hypertension using echocardiography even if they do not have any symptoms of pulmonary artery hypertension. If their systolic pulmonary artery pressure estimated with echocardiography is high, right heart catheterization is recommended [62]. After a diagnosis of porto-pulmonary hypertension, the treatment strategy is based on that of pulmonary artery hypertension.

**Figure 2:**
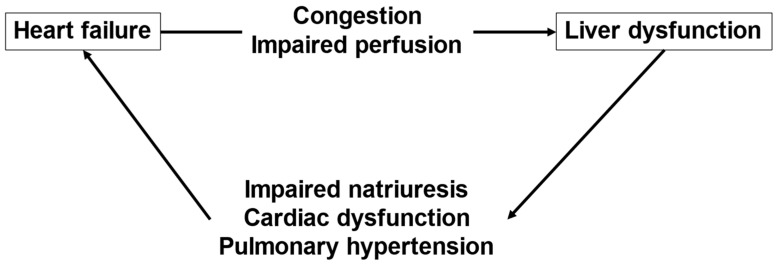
The interaction between heart failure and liver dysfunction.

Figure 2 shows a summary of the interactions between HF and liver dysfunction.

## 4. Medical Therapy for CVD and Liver Damage

Several drugs which are widely used for the treatment of CVD are known to be able to cause liver damage [8]. Amiodarone, which is classified as a class III antiarrhythmic drug, has multiple electrophysiologic properties. Amiodarone has been used for the treatment of life-threatening ventricular arrhythmias and atrial fibrillation in patients with HF. Amiodarone is extensively concentrated in tissues including the liver, which explains its organ-specific adverse effects [63]. Regarding adverse effects on the liver, abnormalities in liver function tests, hepatitis, and cirrhosis may occur. Amiodarone induces liver steatosis histologically resembling alcohol-induced liver injury [64,65]. Amiodarone interferes with oxidative phosphorylation, which causes adenosine triphosphate depletion [66,67]. adenosine triphosphate depletion leads to the reduced activity of the smooth endoplasmic reticulum Ca^2+^ pump, which produces endoplasmic reticulum stress and lipid accumulation [68]. Because amiodarone is lipid-soluble and has a prolonged half-life, the adverse effects can continue for a long duration even after its withdrawal. Thus, its usage needs cautious monitoring including liver function tests. Enalapril, one of the angiotensin-converting enzyme inhibitors that are widely used as a treatment of HF or antihypertension, has been reported to possibly cause liver damage.

Whereas several drugs cause liver damage, there are drug treatments for HF that are reported to improve liver conditions. Sodium-glucose co-transporter-2 inhibitors are oral antidiabetic drugs that inhibit renal proximal tubules from reabsorbing glucose and increase urinary glucose excretion. Sodium-glucose co-transporter-2 inhibitors have some benefits beyond their glucose-lowering effects, such as promoting natriuresis and osmotic diuresis based on glycosuria. These effects advantageously affect patients with HF as they decrease cardiac preload and improve prognosis [69,70,71,72,73,74]. Thus, current guidelines for the management of HF recommend the use of sodium-glucose co-transporter-2 inhibitors for patients with both HF with reduced ejection fraction and HF with preserved ejection fraction regardless of the presence of diabetes mellitus [44]. Because sodium-glucose co-transporter-2 inhibitors have noninsulin-dependent glucose-lowering effects, their advantages as a treatment for diabetes mellitus are also expected for patients with impaired insulin resistance such as obesity [75,76]. For these patients, sodium-glucose co-transporter-2 inhibitors have also been reported to improve liver conditions [77,78]. In patients with NAFLD that is closely related to diabetes mellitus and obesity as above mentioned, sodium-glucose co-transporter-2 inhibitors improve liver function and fibrosis [79,80]. Recently, it was reported that sodium-glucose co-transporter-2 inhibitors might improve liver fibrosis even in patients with NAFLD without diabetes mellitus [81]. Thus, sodium-glucose co-transporter-2 inhibitors can be an efficient treatment option for patients with NAFLD regardless of the presence of diabetes mellitus, which is similar to HF [82]. Calcium channel blockers, which may be a cause of liver damage as above mentioned, were also indicated to improve liver disorder in previous studies. Verapamil, which is a calcium channel blocker with negative inotropic, chronotropic, and dromotropic effects, is reported to have a protective effect against liver damage. Previous studies showed verapamil reduced inflammation, insulin resistance, and liver steatosis in mice models of high-fat diet-induced obesity [83]. Nifedipine, which is one of the representative calcium channel blockers as antihypertensive drugs, is also reported to improve liver damage. In a previous report, nifedipine decreased fibrosis and the serum level of aspartate aminotransferase by upregulating the peroxisome proliferator-activated receptor-γ receptor in rats with NAFLD induced by an L-methionine and choline-deficient diet [84]. Although these medication therapies have not been established in patients with comorbidity of both CVD and liver disease, it may be expected that they can be optional treatment strategies to prevent the development of CVD and improve their prognosis.

## 5. Clinical Applications

### 5.1. NAFLD Management and the Risk of CVD

NAFLD has a close relationship with ASCVD. In patients with NAFLD, the degree of liver fibrosis has a significant association with cardiovascular prognosis [85]. The gold standard for the evaluation of liver fibrosis is liver biopsy. However, a biopsy is an invasive procedure as a screening test [86,87]. As a non-invasive assessment, several biomarkers are used. The fibrosis-4 index (FIB-4 index) and the NAFLD fibrosis score (NFS) are widely used non-invasive scoring systems for the evaluation of liver fibrosis [88,89]. The FIB-4 index score is calculated as follows: FIB-4 index score = (age (years) × aspartate aminotransferase (U/L))/(platelet count (10^9^/L) × alanine aminotransferase ^1/2^ (U/L)) [90]. NFS is calculated as follows: NFS = −1.675 + 0.037 × age (years) + 0.094 × body mass index (kg/m^2^) + 1.13 × impaired fasting glucose/diabetes (yes = 1; no = 0) + 0.99 × aspartate aminotransferase/alanine aminotransferase ratio − 0.013 × platelet count (10^9^/L) − 0.66 × serum albumin (g/dL) [88]. These calculations are described in the Table 1, and they reflect the predicted risk of ASCVD [88,91]. In previous reports, the cut-offs for these scores to predict liver fibrosis and the risk of ASCVD in patients with NAFLD were <1.45 and >3.25 or ≥2.67 for the FIB-4 index and <−1.455 and >0.676 for NFS [88,91].

Liver stiffness measured with shear wave elastography has been used to evaluate liver fibrosis non-invasively [43,44,45,46,47,48,49,50,51,52,53,54,55,56,57,58,59,60,61,62,63,64,65,66,67,68,69,70,71,72,73,74,75,76,77,78,79,80,81,82,83,84,85,86,87,88]. A shear wave is generated by inducing a push pulse of an ultrasound wave, which deforms a part of the tissue. The velocity of the shear wave within the tissue that is detected by tracking the pulse can evaluate the elasticity of the shear wave, which is related to the organic stiffness. Liver stiffness also has an association with the histological fibrosis stage in patients with liver disease [43]. Liver stiffness is also associated with left ventricular diastolic dysfunction in patients with NAFLD [92]. Thus, NAFLD patients with high scores for the scoring indices or liver stiffness should be investigated to determine whether they have CVD. Indeed, NAFLD patients, especially patients with high scores for fibrosis indices or liver stiffness, also have high coronary artery calcium scores, which is an established marker of ASCVD assessed with computed tomography [93,94,95,96]. Patients with NAFLD tend to have high risks of plaque development and stenosis in coronary arteries compared with patients without NAFLD based on coronary computed tomography angiography (CCTA) [97]. The presence of NAFLD also has incremental prognostic value over the CCTA findings in patients with clinically suspected coronary artery disease [98,99]. These findings indicate that NAFLD is associated with the risk of ASCVD, and NAFLD itself, could be a risk factor for cardiovascular disease.

In addition, as mentioned above, the pathology of NAFLD and inflammation have a known relationship. Many inflammatory molecular biomarkers have a close link to the development of NAFLD. Each of these biomarkers also shares a relationship with the development of NAFLD comorbidities, such as diabetes mellitus, obesity, metabolic syndrome, and CVD [100]. Among them, tumor necrosis factor-α, interleukin-8, interleukin-10, plasminogen activator inhibitor 1, sterol regulatory element binding protein-1c, and apoB are especially reported for their association with the development of CVD. Although the clinical roles of inflammatory assessment for the management of CVD have not been established, these molecular biomarkers may also be efficient markers for liver fibrosis and liver stiffness as well.

Recently, pericoronary adipose tissue attenuation on CCTA was higher in patients with vs. without NAFLD, and patients with NAFLD with higher pericoronary adipose tissue attenuation had worse cardiovascular prognoses [101,102]. Pericoronary adipose tissue attenuation is a novel method for the risk assessment of cardiac mortality, reflecting pericoronary inflammation [103]. Considering these findings, high levels of vascular inflammation might play a role in the progress of CVD in patients with NAFLD. Thus, CCTA is a feasible modality for the evaluation of CVD in patients with NAFLD. In addition, computed tomography is useful for not only risk assessment but also the diagnosis of NAFLD through the measurement of hepatic and splenic computed tomography attenuation. The Hounsfield Unit attenuation of liver on computed tomography scans decreased in patients with NAFLD reflecting the presence of fat, which shows a low degree of the Hounsfield Unit attenuation in liver. Previous reports showed that a Hounsfield Unit attenuation of liver <40 Hounsfield Unit indicates >30% of liver fat content [104]. Because the Hounsfield Unit attenuation of liver is higher than that of the spleen as usual, reversed liver-to-spleen ratio also indicates high liver fat deposition. Thus, the liver-to-spleen ratio in the Hounsfield Unit <1.0 can be also used for the diagnosis of NAFLD non-invasively [105].

Therefore, patients with NAFLD should be evaluated to determine the degree of liver fibrosis using the FIB-4 index, NFS, and shear wave elastography. If the values are high, CVD screening should be performed initiatively, especially CCTA, for patients with symptoms indicating CVD, such as chest pain.

### 5.2. Assessment of Liver Fibrosis as a Prognostic Marker for HF

Because HF and liver conditions interact, the evaluation for liver disorders is efficient for management in patients with HF. Thus, the current guidelines for the management of HF recommend laboratory evaluation including liver function tests in patients who are diagnosed as presenting with HF to optimize management [44]. The biomarkers which indicate liver disorder relate to the worse prognosis in patients with HF. Serum levels of aminotransferase, such as aspartate aminotransferases and alanine aminotransferase, and biliary enzymes, such as g-glutamyl transpeptidase, alkaline phosphatase, and bilirubin, have been reported to be prognostic factors in patients with HF. Among them, previous reports implicated that the aminotransferases especially had an association with the clinical signs of hypoperfusion and the biliary enzymes had an association with the signs of systemic congestion and elevated right-sided filling pressure [49,106]. These findings indicate that impaired liver perfusion relates to elevated levels of aminotransferase and liver congestion relates to elevated levels of biliary enzymes, respectively.

As mentioned, liver stiffness measured with shear wave elastography has been used to evaluate liver fibrosis non-invasively [107]. Hepatic elasticity is reported to increase with progressive HF stages [44]. In HF patients, liver stiffness has been reported to reflect central venous pressure and right ventricular dysfunction and has also been reported to be associated with HF progression [108,109,110]. Additionally, high liver stiffness is associated with a worse prognosis in patients with HF [110,111]. Thus, the assessment of liver stiffness could be considered a surrogate marker for prognosis in patients with HF. However, shear wave elastography requires special equipment and is limited in the presence of obesity or ascites [109]. As an alternative non-invasive assessment of liver fibrosis, the FIB-4 index and NFS are especially related to liver fibrosis and are widely used in patients with liver disease [88,89]. These indices have also recently been investigated as prognostic surrogate markers in patients with HF. The FIB-4 index reflects the future development of HF and is associated with prognosis in HF [112,113,114,115]. The FIB-4 index has a prognostic impact regardless of the HF classification by ejection fraction. This is so even in patients with HF with preserved ejection fraction, whose level of natriuretic peptides tends to be significantly lower than that in patients with HF with reduced ejection fraction [114,116,117,118]. The prognostic impact of NFS in patients with HF has also been reported [119]. However, the optimal cut-offs of the FIB-4 index and NFS scores to predict worse prognosis in patients with HF have not been established. However, these assessments of liver condition are feasible for the management of HF. Additionally, liver stiffness measured with shear wave elastography and the FIB-4 index can be used to predict cardiovascular prognosis in patients with significant tricuspid regurgitation, which can cause systemic venous congestion [120,121]. These indices are also associated with right ventricular dysfunction and central venous or right atrial pressure in patients with HF [109,110,114,115]. Furthermore, these non-invasive assessments for liver fibrosis are also efficient in order to predict the presence of Fontan-associated liver disease, which is a specific congestive hepatopathy occurring in patients after the Fontan procedure and one of the important comorbidities relating to their worse prognosis as above mentioned. These findings indicate that liver stiffness measured with shear wave elastography and the FIB-4 index may be useful surrogate markers for prognosis, especially in right-sided heart failure, as a reflection of liver congestion.

Natriuretic peptide concentrations are initially low in patients with HF owing to causes upstream from the left ventricle, such as right-sided heart failure, because of the absence of a significant increase in left ventricular wall stress [122]. In these situations, the measurement of the FIB-4 index, instead of or in addition to natriuretic peptides, might be a feasible alternative for the assessment of prognosis in patients with HF.

## 6. Conclusions

In conclusion, CVD and liver disease interact with each other, and the evaluation of both diseases is important. It may be sometimes difficult to distinguish their interaction and confounding effects between them in a clinical setting because these diseases share similar risk factors and basic pathologies. Although this limitation remains, the importance of both CVD and liver disease has been increasing in the current era of ageing populations. Especially, early detection and prevention of CAD are thought to be important, considering its high impact on health prognosis. Non-invasive and convenient approaches for the assessment of liver conditions such as the FIB-4 index may be efficient for daily practice to help the management of CVD. Thus, cardiologists must have a knowledge of liver diseases to understand the pathologies and risks associated with CVD. However, whether management and interventions for liver disease improve the prognosis of CVD has not been fully understood. As above mentioned, several treatments, such as diet therapy and medication therapy, including sodium-glucose co-transporter-2 inhibitors, are reported to possibly improve the liver condition. In addition, several anti-fibrotic drugs are currently under investigation [123,124]. As well as the recent progression of treatment for CVD, the novel treatment for liver disease may advance in the near future. It is expected that these treatment strategies considering favorable effects for liver disease can prevent the development of CVD in patients with liver disease or can improve the prognosis in patients with CVD who have commodities of liver disease. Future investigations are needed to obtain a greater perspective.

## Figures and Tables

**Table 1:** The calculation of the FIB-4 index and NFS.

Indices	Equation
FIB-4 index	(Age(years) × AST(U/L))/(platelet (10^9^) × ALT^1/2^ (U/L))
NFS	−1.675 + 0.037 × age (years) + 0.094 × body mass index (kg/m^2^) + 1.13 × impaired fasting glucose/diabetes (yes = 1; no = 0) + 0.99 × AST/ALT ratio − 0.013 × platelet count (10^9^/L) − 0.66 × serum albumin (g/dL)

FIB-4 index, fibrosis-4 index; NFS, non-alcoholic fatty liver disease fibrosis score; AST, aspartate aminotransferase; ALT, alanine aminotransferase.

## Data Availability

Not applicable.

## Abbreviations

ASCVD	atherosclerotic cardiovascular disease
CCTA	coronary computed tomography angiography
CVD	cardiovascular disease
FALD	Fontan-associated liver disease
FIB-4 index	fibrosis-4 index
HCC	hepatocellular carcinoma
HDL	high-density lipoprotein
HF	heart failure
HRP	high-risk plaque
LDL	low-density lipoprotein
LV	left ventricular
NAFLD	non-alcoholic fatty liver disease
NFS	non-alcoholic fatty liver disease fibrosis score
PoPH	porto-pulmonary hypertension
RV	right ventricular
TG	triglyceride
